# Treatment of postoperative non-union with internal fixation loosening of Garden IV femoral neck fracture with teriparatide in a young adult: A case report

**DOI:** 10.3389/fsurg.2022.938595

**Published:** 2022-11-03

**Authors:** Lili Lai, Yifan Li, Miaoda Shen, Xuanwei Wang, Cheng Zhong, Sanzhong Xu

**Affiliations:** Department of Orthopedics, The First Affiliated Hospital, Zhejiang University School of Medicine, Hangzhou, China

**Keywords:** teriparatide, fracture nonunion, femoral neck fracture, young adults, case report

## Abstract

**Background:**

Postoperative non-union of femoral neck fracture often needs secondary operation. We report a case of a postoperative non-union of femoral neck fracture treated with teriparatide.

**Case presentation:**

A young male patient with Garden IV femoral neck fracture who showed no obvious signs of healing 3 months after percutaneous hollow nail fixation in which the fracture line was enlarged and the hollow nail was withdrawn. Bone non-union healed after 6 months of continuous subcutaneous injection of teriparatide at a dosage of 20 mg/day after the patient refused a secondary surgery. As far as we know, there have been no relevant reports on this type of fracture yet.

**Conclusions:**

Teriparatide is expected to be beneficial in treating young patients with a displaced femoral neck fracture who have difficulty in healing from non-union and who are keen on avoiding secondary surgery.

## Background

Femoral neck fractures are uncommon in young adults (3%–10%) and are often caused by high-energy trauma. Anatomical reduction and stable internal fixation are the key to the current treatment for young patients with higher activity and functional requirements (1, 2). However, the incidence of delayed union, non-union, and even osteonecrosis of the femoral head after operation is still high (3). Young patients can be treated by osteotomy to enhance the mechanics of machinery or bone grafting to improve the biological environment of non-union regions in order to preserve the femoral head and hip joint once diagnosed as delayed union or non-union (4).

Teriparatide is a recombinant preparation derived from the first 34 amino acids of the human parathyroid hormone (rhPTH). It has been approved by the United States Food and Drug Administration (USFDA) for the treatment of male osteoporosis (5, 6). There has been some evidence that daily or weekly subcutaneous injection of teriparatide can significantly improve the non-union of the long bone shaft (7), sternum (8), metatarsals (9), and so on. In this report, we introduce a case of non-union with a loosening of internal fixation screws in a young patient with femoral neck fracture. The fracture was healed successfully by daily subcutaneous injection of teriparatide, following which the internal fixation screws were removed.

## Case presentation

**Figure 1:**
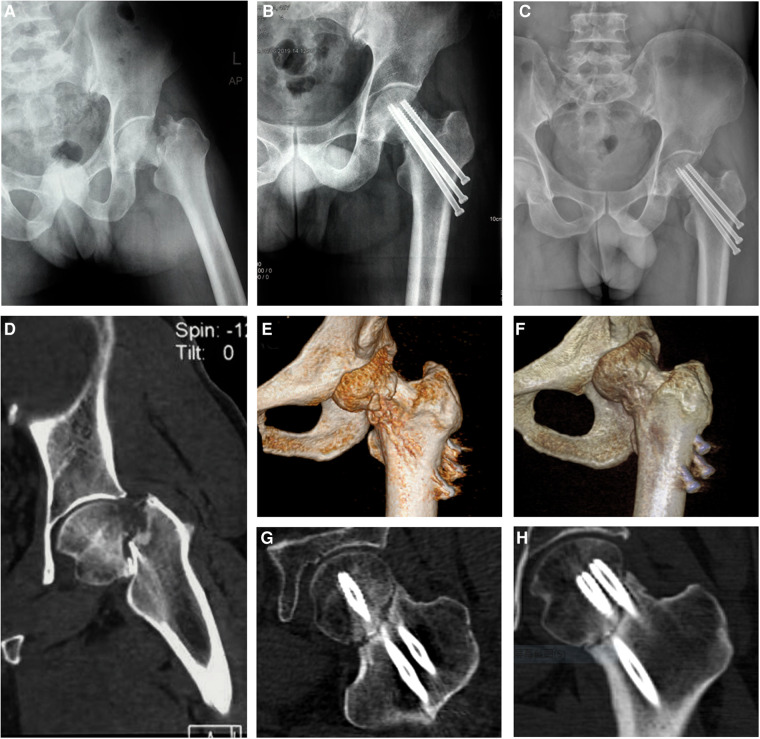
X--ray (**A**) and CT (**D**) showing a Garden IV fracture of the left femoral neck. The x-ray (**B**) and CT (**E,G**) showing bone resorption at the fracture site with internal fixation loosening and slipping 8 weeks after operation. The x-ray (**C**) and CT (**F,H**) showing the non-union of fracture 12 weeks after operation when the patient first visited our hospital and was started on a subcutaneous injection of teriparatide.

**Figure 2:**
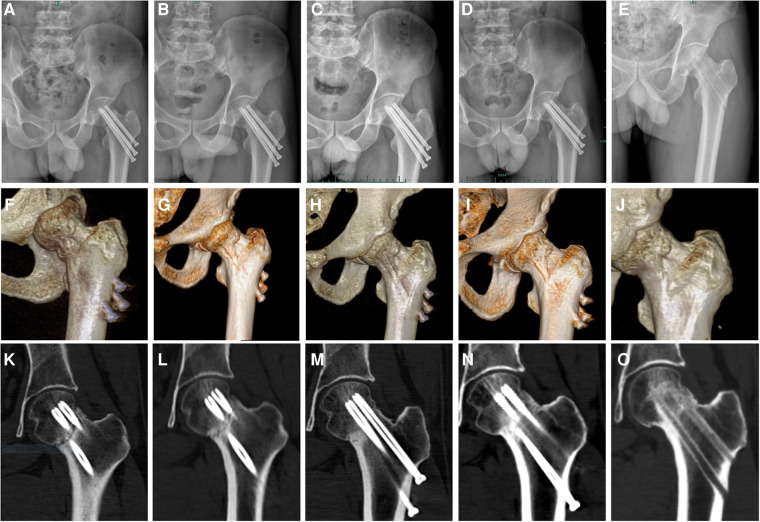
Radiological images (**A, F, K**) showing trabeculae bridging through fracture sites and a reduction of the fracture space after 3 months of taking the drug. Representative images of the patient regularly reviewed while taking medication (**B, G, L**). The x-ray (**C**) and CT film (**H, M**) showing the bone healing completely after 6 months of stopping the drug. Representative images of the patient regularly reviewed after drug withdrawal (**D, I, N**). Internal fixation screws were removed in July 2021 (**E, J, O**) when the patient returned to normal activity feeling no pain.

**Figure 3:**
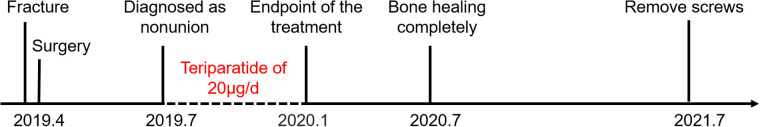
The treatment timeline of this patient.

A 45-year-old male patient sustained a Garden IV fracture of the left femoral neck (Figures 1A,D) caused by a fall and underwent cannulated hip screw surgery at a local hospital in April 2019. Medical history revealed no diabetes mellitus, chronic disease, hypertension, smoking, alcoholism, or long-term medication. The patient was followed up every 4 weeks with x-ray and three-dimensional computed tomography (CT) examinations. The x-ray (Figure 1B) and CT (Figures 1E,G) still showed bone resorption at the fracture site with internal fixation loosening and slipping 8 weeks after operation. After experiencing local swelling and soreness in the affected area, the patient consulted with the Department of Orthopedics of our hospital on July 2019 (12 weeks after operation) . The x-ray (Figure 1C) and CT (Figures 1F,H) images showed poor fracture healing, screw loosening, and slipping. Arthroplasty was recommended by the doctor of the hospital where the patient had first consulted. Physical examinations showed no redness, local heat, swelling, and pain. Laboratory examinations including those for determining the erythrocyte sedimentation rate (ESR), C-reactive protein (CRP), as well as white blood cell (WBC) count with their normal ranges, were performed to exclude postoperative infection and infectious bone non-union. A series of laboratory examinations, including parathyroid hormone (PTH), serum alkaline phosphates, calcium, phosphorus, creatinine, and 25-(OH)_2_D_3_ within the normal ranges, were performed to exclude possible metabolic disorders. A dual-energy x-ray bone mineral density test indicated normal bone mineral density. Finally, the patient was diagnosed with non-union and he consented to empirical, off-label therapy with the approved dose of teriparatide (20 μg/day for osteoporosis) instead of a second surgery. Trabeculae bridging through fracture sites and fracture space reduction were seen on the radiological images (Figures 2A,F,K) after 3 months of the patient taking the drug. No side effects of the drug or any abnormal laboratory test value were observed throughout the 6-month treatment regimen. It is worth mentioning that the value of Procollagen type I N-terminal propeptide (P1NP) increased significantly (75.57–165.7 µg/L) during the period of medication, which could be seen as a sign of bone healing (10). X-ray (Figure 2C) and CT film (Figures 2H,M) showed the bone healing completely after 6 months of stopping the drug. The internal fixation screws were removed in July 2021 (Figures 2E,J,O) when the patient returned to normal activity feeling no pain. The treatment timeline of the case is showed in Figure 3.

## Discussion

Arthroplasty is generally considered to be the most effective treatment for displaced femoral neck fractures in elderly patients; however, for younger patients, open reduction and internal fixation is considered the gold standard approach (11). Postoperative bone non-union is common, and the related risk factors usually include inaccurate reduction, unstable fixation, infection, diabetes mellitus, alcoholism, smoking, osteoporosis, diet (low calcium, vitamin D), and so on (12, 13). It is generally recognized that the diagnostic criterion of delayed union is no fracture healing after 6 months. Meanwhile, non-union is defined as no fracture healing for at least 9 months, and bone healing can be achieved only by effecting a change in treatment after diagnosis (14, 15). However, sometimes there is no necessity to wait for such a long time when there is no progress in callus formation at the fracture site at a 4-week follow-up interval (16, 17). Mark Jackson et al. suggested that fixation failures may speed up the diagnosis, and early diagnosis of impending problems is possible. A careful examination of plain radiographs 3 months after fracture could predict failures (18). Our patient complained of leg pain with inability to walk. Radiological examinations showed that there was no obvious callus formation at the fracture site of the affected limb, and the internal fixation hollow nail showed loosening and withdrawal. Therefore, we believe that the diagnosis of non-union should be timely and based on the fracture type, postoperative time, clinical manifestations, and radiological manifestations, so as to avoid delaying treatment and frittering any such opportunity.

The native hip joint should be preserved as far as possible in the treatment of femoral neck non-union for physiologically young patients (19). There are limited surgical options available to the orthopedic surgeon including revision of internal fixation with autogenous bone grafting, angulation osteotomy, and vascularized bone grafting for the treatment of femoral neck non-union with preservation of the femoral head (20). However, no technique has been proved to give a completely satisfactory result, with even functional injuries or complications occurring because of secondary surgical intervention.

Some cases related to the treatment of fracture non-union with teriparatide are now being reported (9, 21, 22). Kastirr et al. (9) included 32 patients with non-union treated with teriparatide in a prospective study, and 95% of the fractures healed completely after treatment in these patients. However, patients with a non-union of femoral neck fractures were not involved in the study. Lee et al. (21) reported a case of a postoperative non-union of femoral neck fracture in a young man treated with teriparatide. However, it was not known whether there was displacement, and callus formation could be found at the fracture site from the x-ray and CT provided. Mitani et al. (22) reported a case of a steroid-induced femoral neck fracture non-union in an elderly rheumatoid patient, which was successfully healed by a weekly treatment of 56.5 μg teriparatide. This case was different from our case, and the time of use of teriparatide was not the same. Our patient showed a non-union of the femoral neck and loosening of the hollow nail 3 months after percutaneous hollow nail fixation of the femoral neck, which was distinct from all reported cases previously. The patient refused to undergo revision surgery and received treatment at a dose of 20 µg/day for 6 months, and there was regular reexamination of the x-ray, CT, and blood biochemical indices such as bone turnover markers. As far as we know, this is the first case of a successful treatment of non-union of a displaced femoral neck fracture with teriparatide in an adult.

In summary, teriparatide could be used in the treatment of non-union of displaced femoral neck fractures in young patients who are keen on avoiding secondary surgery on the basis of our case and treatment experience. Therefore, additional randomized clinical studies are necessary to clarify the effectiveness and scope of application of the drug.

## Data Availability

The original contributions presented in the study are included in the article/Supplementary Material, further inquiries can be directed to the corresponding author.
